# NMR studies on lignocellulose deconstructions in the digestive system of the lower termite *Coptotermes formosanus* Shiraki

**DOI:** 10.1038/s41598-018-19562-0

**Published:** 2018-01-22

**Authors:** Didi Tarmadi, Yuki Tobimatsu, Masaomi Yamamura, Takuji Miyamoto, Yasuyuki Miyagawa, Toshiaki Umezawa, Tsuyoshi Yoshimura

**Affiliations:** 1 Research Institute for Sustainable Humanosphere (RISH), Kyoto University, Gokasho Uji, Kyoto, 611-0011 Japan; 2 Research Center for Biomaterials, Indonesian Institute of Sciences (LIPI), Jl. Raya Bogor KM.46, Cibinong, Bogor, West Java, 16911 Indonesia; 3 Research Unit for Development and Global Sustainability, Kyoto University, Gokasho, Uji, Kyoto, 611-0011 Japan

## Abstract

Termites represent one of the most efficient lignocellulose decomposers on earth. The mechanism by which termites overcome the recalcitrant lignin barrier to gain access to embedded polysaccharides for assimilation and energy remains largely unknown. In the present study, softwood, hardwood, and grass lignocellulose diets were fed to *Coptotermes formosanus* workers, and structural differences between the original lignocellulose diets and the resulting feces were examined by solution-state multidimensional nuclear magnetic resonance (NMR) techniques as well as by complementary wet-chemical methods. Overall, our data support the view that lignin polymers are partially decomposed during their passage through the termite gut digestive system, although polysaccharide decomposition clearly dominates the overall lignocellulose deconstruction process and the majority of lignin polymers remain intact in the digestive residues. High-resolution NMR structural data suggested preferential removal of syringyl aromatic units in hardwood lignins, but non-acylated guaiacyl units as well as tricin end-units in grass lignins. In addition, our data suggest that termites and/or their gut symbionts may favor degradation of C–C-bonded β–5 and resinol-type β–β lignin inter-monomeric units over degradation of ether-bonded β–*O*–4 units, which is in contrast to what has been observed in typical lignin biodegradation undertaken by wood-decaying fungi.

## Introduction

Lignin, a heterogeneous phenylpropanoid polymer derived primarily from oxidative couplings of *p*-hydroxycinnamyl alcohols (monolignols) and their derivatives, is a key component of the lignocellulose produced in the secondary cell walls of vascular plants, where lignin encrusts cell-wall polysaccharides (i.e., cellulose and hemicelluloses) and provides them with increased mechanical strength, imperviousness, and resistance to pathogens^1^. Due to its chemical complexity and general lack of vulnerable linkages, lignin confers lignocellulose with high resistance to most forms of microbial attack. Lignin biodegradation is thus a key step for lignocellulose-oriented carbon recycling in terrestrial ecosystems. The process has also been a major research focus motivated by potential biotechnological applications in plant biomass utilization. In this context, lignin biodegradation via wood-decaying fungi, known as white-rot and brown-rot decay, has been extensively studied^2–6^. However, lignin degradation processes in more complex ecosystems such as those undertaken by wood-feeding insects remain largely elusive.

Termites consume 3–7 billion tons of lignocellulosic materials annually and thereby represent one of the most prolific and efficient lignocellulose decomposers on earth^7^. While this ability to digest lignocellulose makes them a notorious pest of wooden structures, they also have been recognized as efficient “bioreactors” and a potential source of biomass-processing enzymes for the production of biofuels and biomaterials from plant biomass^7–12^. The digestion of cell wall polysaccharides by termites is a highly coordinated process achieved by the termite host and its gut-resident microbial symbionts. In so-called lower termites harboring flagellated protists in their hindguts, the degradation of cellulose is effectively achieved with the aid of a variety of cellulolytic enzymes supplied by hindgut protists, whereas in so-called higher termites lacking gut protists, diverse prokaryotic gut symbionts as well as the occasional ectosymbiotic fungi, support an efficient cellulolytic process^12^.

Lignin deconstruction is a critical step in lignocellulose digestion as it first enables dissociation of the recalcitrant lignin polymers from the cellulose and hemicelluloses in which they are embedded, making the polysaccharides available for assimilation and energy. Although the degradation of cell-wall polysaccharides in the termite digestive system has been relatively well documented in the literature, it is yet largely unclear how termites overcome the lignin barrier to gain access to them. It had long been thought that lower termites and their gut symbionts have little or no ability to decompose natural lignin polymers^8,13^. Although several reports documented that gut flora of wood-feeding lower termites exhibited *in vitro* and/or *in vivo* abilities to modify monomeric and dimeric lignin-associated aromatic molecules^14–16^, earlier structural studies on polymeric lignins using conventional wet-chemical and spectroscopic approaches detected no conclusive evidence for chemical modification of the polymers upon their passage through the gut of lower termites^17^. However, more recent studies, especially those using advanced thermochemolysis, such as pyrolysis-gas chromatography/mass spectrometry (Py-GC/MS) with or without *in situ* derivatization with tetramethylammonium hydroxide (TMAH), have provided data supportive of chemical modifications of lignin polymers in the gut digestive system of lower termites; Py-GC/MS of digested lignocelluloses detected characteristic pyrolytic fragments conceivably derived from degraded lignin polymers with aromatic ring modifications and/or side-chain oxidations^18–21^. As such, thermochemolysis is indeed a versatile tool to characterize lignins with high sensitivity^22–26^. However, since the method is not fully quantitative, it is yet unclear how abundantly such lignin processing occurs in the termite digestive system.

On another front, solution-state multi-dimensional NMR spectroscopy has proven to be highly effective in characterizing the chemical structures of various lignocellulosic materials. Such NMR techniques have been increasingly applied to understanding lignocellulose biodegradation processes such as those undertaken by white-rot and brown-rot fungi^27–30^ as well as by a fungus-cultivating higher termite, *Odontotermes formosanus* Shiraki^31^, for whom the detailed chemical changes in the complex polysaccharide and lignin polymer structures were successfully tracked. In the present study, we applied these NMR techniques to study lignocellulose decomposition in the gut digestive system of a lower termite *Coptotermes formosanus* Shiraki, one of the most destructive and economically important wood-feeding termites in the world^32^. To investigate the actions of the *C. formosanus* digestive system on different types of lignocellulose substrates, we fed *C. formosanus* workers three different lignocellulose diets prepared from softwood (Japanese cedar), hardwood (Japanese beech), and grass (rice straw). Structural differences between the original diets and the resulting feces were closely examined by solution-state ^1^H–^13^C short-range correlation (HSQC) NMR as well as by complementary wet-chemical methods. We discuss the obtained structural data with special emphasis on the fate of lignin polymers in the termite gut digestive system.

## Results

### Termite survival and mass loss of lignocellulose

**Figure 1:**
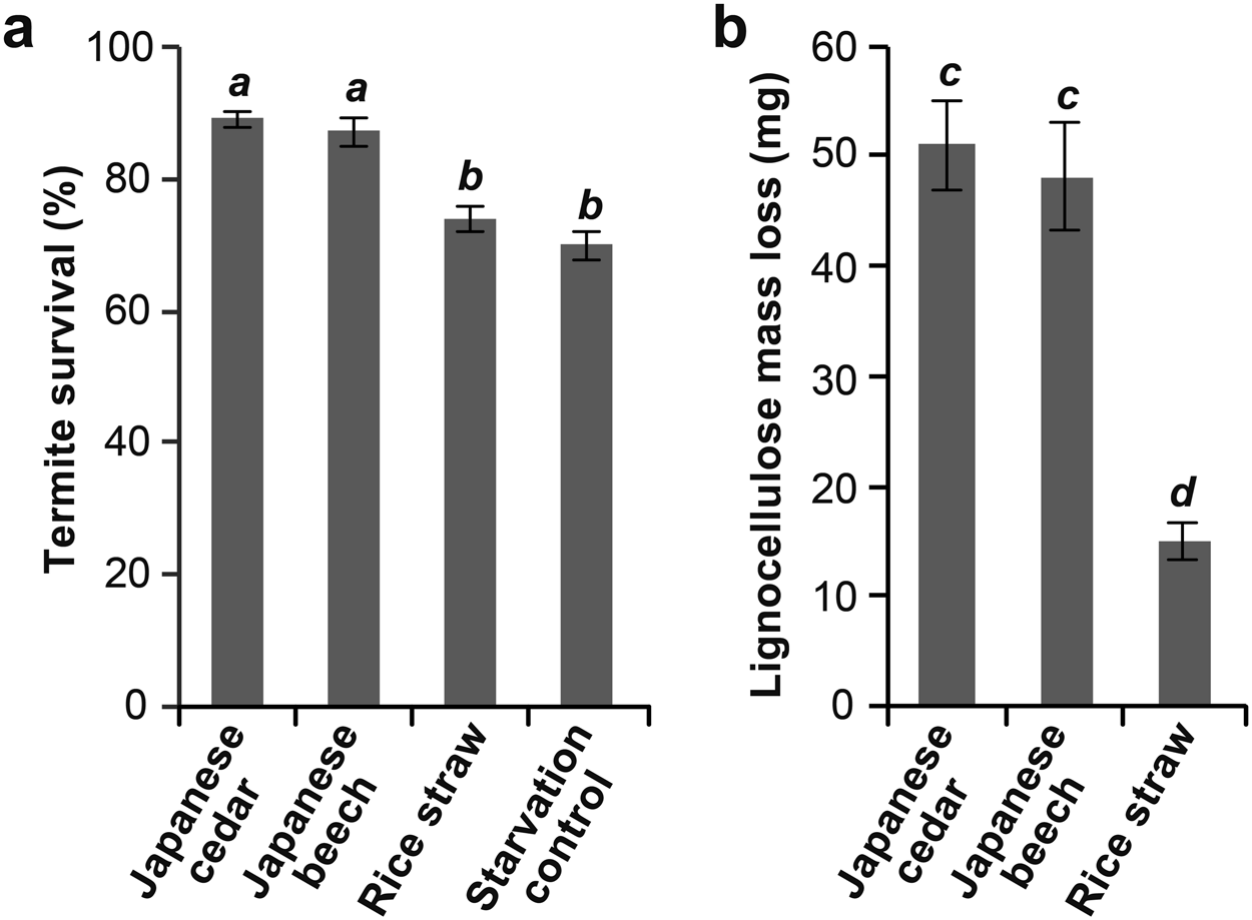
Termite survival (**a**) and lignocellulose mass loss (**b**) after 3-week feeding by *C*. *formosanus* termite workers on Japanese cedar (softwood), Japanese beech (hardwood), and rice straw (grass) lignocellulose diets. Values with the same letter are not significantly different (Tukey HSD test; *P* < 0.05; n = 3) following one-way ANOVA. Error bars represent standard deviations.

At the onset of this study, we first observed the termite survival and dietary mass consumed for *C*. *formosanus* workers fed lignocellulose diets. The survival rates of workers fed J. cedar (softwood) and J. beech (hardwood) diets after 3 weeks of feeding were similar (~90%) and significantly higher than those recorded for workers fed rice straw (grass) diet (~74%) and starvation controls (~70%) (Fig. 1a). Our statistical analysis failed to detect any significant difference between the workers fed on rice and the starvation control. Furthermore, it was suggested that *C*. *formosanus* workers consumed significantly less of the rice grass diets than the J. cedar and J. beech wood diets (Fig. 1b). Our data collectively suggest that the rice grass diet was less nutritious for *C*. *formosanus* workers compared to the softwood and hardwood diets. The result is in line with our previous termite feeding study^33,34^, and also with the observation that lower termites including *C*. *formosanus* generally prefer wood to grass for feeding in nature^12^.

### Chemical compositional analysis of lignocelluloses digested by *C*. *formosanus* workers

Digested lignocellulose samples (feces) from *C*. *formosanus* workers were first characterized by lignocellulose compositional analysis using wet chemical methods. Sugar and lignin content analyses determined an approximately 50% proportional decrease in cellulosic crystalline glucan and a 90% proportional increase in lignin content in the digested softwood J. cedar lignocellulose (Table 1). While mannan was moderately (22%) depleted, the other major hemicellulosic sugars, i.e., xylan, arabinan, and galactan, were significantly enriched along with lignin in the digested J. cedar lignocellulose. The result suggests the selective digestion of cellulose over hemicelluloses and lignins in the *C*. *formosanus* digestive system. In the digestion of J. beech hardwood, an approximately 50% proportional decrease and 70% proportional increase in cellulose and lignin content, respectively, were likewise recorded (Table 1). Unlike in the digestion of J. cedar, however, we observed significant decreases in amorphous glucan (~60%), xylan (~74%), and mannan (~89%) in the digestion of J. beech lignocellulose. In the digestion of grass rice straw, we observed relatively moderate changes in both cellulose (~22% decrease) and lignin (~37% increase) content (Table 1). As with J. beech, in the rice lignocellulose digestion we observed significant decreases in the major hemicellulosic sugars such as amorphous glucan (~18%), xylan (~49%), and arabinan (~89%), but enrichments in mannan and galactan. Overall, our data are in line with previous studies reporting preferential conversions of cell-wall polysaccharides over lignins in the termite digestive system. It is most likely that polysaccharide conversions in the *C*. *formosanus* digestive system are highly selective for cellulose degradation, especially in softwood lignocellulose, but expand to include hemicellulosic sugar conversions in hardwood and grass lignocelluloses.

**Table 1:** Chemical analysis of lignocellulose samples digested by C. *formosanus* termite workers

	Japanese cedar		Japanese beech			Rice straw	
	Original	Digested	Original	Digested		Original	Digested
Lignin content (mg/g CWR)	239.2 ± 2.0	**463.0 ± 22.2** ^*^	196.0 ± 3.7	**334.9 ± 2.8** ^*^		150.2 ± 4.7	**205.3 ± 3.0** ^*^
Lignin composition							
Syringyl units, S (%)	N.D.	N.D.	75.2 ± 0.1	**70.2 ± 1.3** ^*^		43.6 ± 1.5	**54.7 ± 0.3** ^*^
Guaiacyl units, G (%)	99.5 ± 0.1	99.8 ± 0.1	24.8 ± 0.1	**29.7 ± 1.3** ^*^		52.3 ± 1.4	**41.9 ± 0.6** ^*^
*p*-Hydroxyphenyl units, H (%)	0.5 ± 0.1	0.2 ± 0.1	trace	0.2 ± 0.1		4.2 ± 0.5	3.5 ± 0.4
S/G ratio	0.00 ± 0.00	0.00 ± 0.00	3.05 ± 0.01	**2.37 ± 0.14** ^*^		0.84 ± 0.05	**1.31 ± 0.03** ^*^
Carbohydrate content (mg/g CWR)							
Glucan (crystalline)	442.1 ± 8.7	**210.7 ± 10.5** ^*^	428.2 ± 40.9	**235.5 ± 23.4** ^*^		454.4 ± 18.5	**354.1 ± 52.0** ^*^
Glucan (Amorphous)	27.2 ± 1.2	27.4 ± 4.2	23.9 ± 2.6	**9.6 ± 1.3** ^*^		22.6 ± 1.2	**18.7 ± 0.6** ^*^
Xylan	64.4 ± 3.2	**108.7 ± 9.5** ^*^	305.82 ± 12.6	**80.2 ± 3.2** ^*^		81.0 ± 5.5	**41.3 ± 1.1** ^*^
Mannan	66.4 ± 2.8	**52.0 ± 4.9** ^*^	8.67 ± 0.6	**1.85 ± 0.7** ^*^		3.4 ± 0.7	**8.3 ± 0.6** ^*^
Arabinan	15.3 ± 0.1	**26.2 ± 2.6** ^*^	7.6 ± 0.3	7.83 ± 1.9		29.8 ± 0.6	**19.5 ± 0.2** ^*^
Galactan	16.1 ± 1.1	**22.3 ± 1.9** ^*^	8.8 ± 0.8	10.9 ± 1.5		12.9 ± 1.4	**15.9 ± 0.6** ^*^

Lignin composition was determined by analytical thioacidolysis. Values are means ± SD (*n* = 3) and asterisks (*) indicate significant difference between original and digested lignocellulose diets (*P* < 0.05). N.D., not detected.

### Cell wall NMRs of lignocelluloses digested by *C*. *formosanus* workers

We then employed 2D NMR techniques to obtain further detailed chemical information of the lignocellulose diets digested by *C*. *formosanus* workers. One of the biggest advantages of current plant cell wall NMR techniques is the ability to analyze an entire cell wall fraction via direct dissolution/swelling methods^35–37^. In the present study, we analyzed whole cell wall fractions of the original and digested lignocelluloses by simply swelling them in DMSO-*d*_6_/pyridine-*d*_5_ (4:1, v/v) after fine ball-milling. This approach provides a global picture of the chemical composition and structure of cell-wall polysaccharides as well as lignins, although highly crystalline cellulose contents tend to be underestimated due to its incomplete gelation^36,37^.

The short-range ^13^C–^1^H correlative (HSQC) NMR spectra of cell walls in the original lignocellulose diets revealed prominent structural differences between the three major types of lignocellulose in nature. As revealed by the aromatic signals in the HSQC spectra (δ_C_/δ_H_, 150–100/8.5–6.0 ppm), lignins in the J. cedar lignocellulose diet follow the pattern of typical softwood lignin, composed of only G units (Figure S1), whereas lignins in the hardwood J. beech and grass rice straw diets are mixtures of G and S units (Figures S2 and S3). As is typical for grass lignocellulose, the spectrum of rice cell walls additionally displayed intense signals from *p*-coumarate^38^ and tricin residues^39,40^ attached mainly on lignins, as well as signals from ferulates mainly on arabinoxylan hemicellulose residues^38^. In the polysaccharide anomeric regions (δ_C_/δ_H_, 110–90/6.0–3.5 ppm), anomeric signals from abundant hemicellulosic polysaccharides, such as glucomannans, glucuronoxylans, and glucuronoarabinoxylans, are visible in J. cedar, J. beech, and rice straw cell wall spectra, respectively (Figures S1, S2, and S3, Table S1). The aliphatic-oxygenated regions (δ_C_/δ_H_, 90–45/6.0–3.5 ppm) are overwhelmed by intense and overlapping polysaccharide contours but they also display well-resolved contours from some of the major lignin side-chains as well as those from acetylated hemicelluloses (Figures  S1,  S2, and  S3, Table S1).

**Figure 2:**
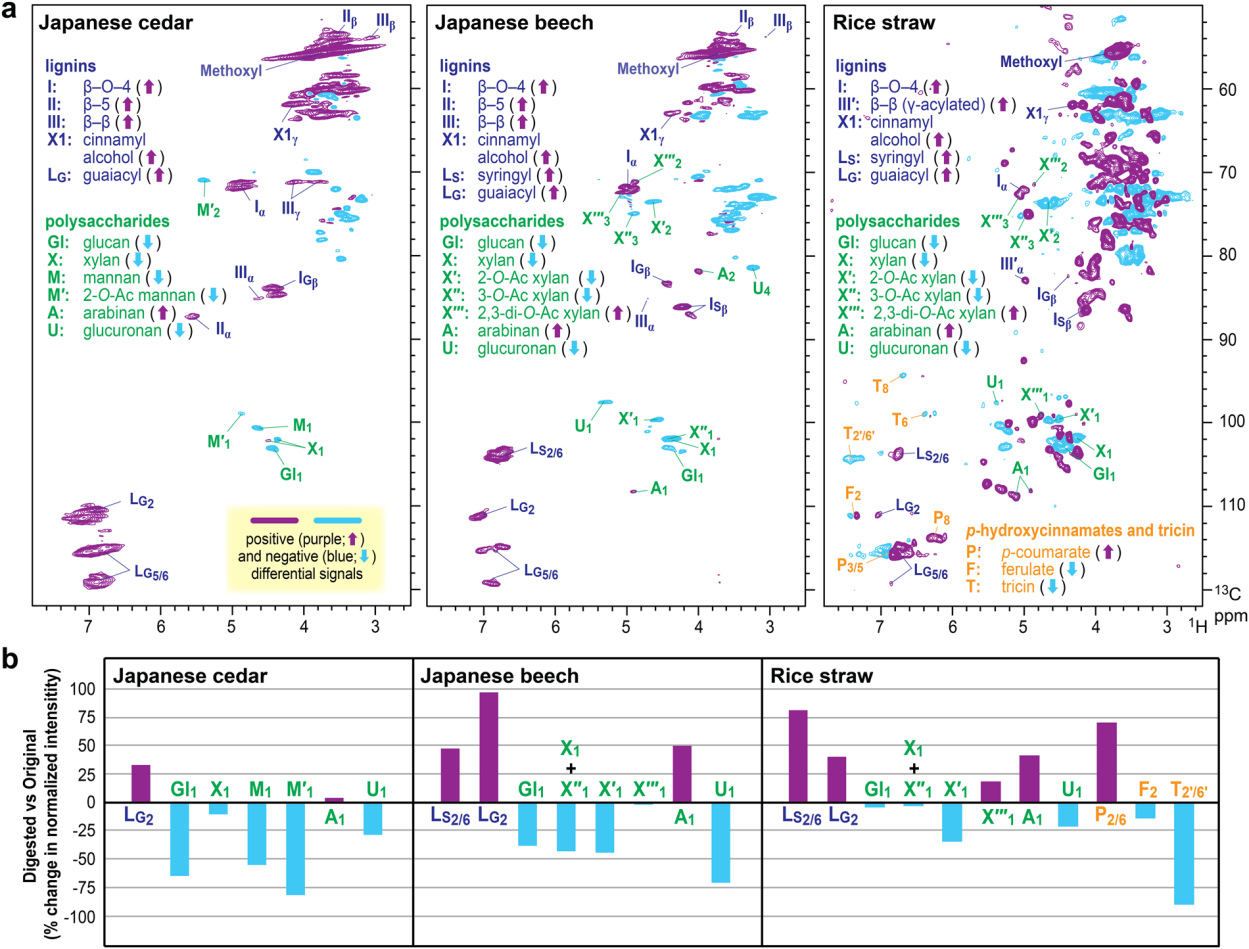
Whole cell wall NMR analysis on original and digested Japanese cedar (softwood), Japanese beech (hardwood), and rice straw (grass) lignocellulose diets fed to *C*. *formosanus* termite workers. (**a**) Differential 2D ^1^H–^13^C correlation (HSQC) spectra for digested versus original cell walls. (**b**) Changes in relative signal abundances determined for major lignin aromatic and polysaccharide anomeric signals appearing in the original and digested cell wall spectra. For signal assignments and structure abbreviations, also see Table S1.

Details on the differences between the original and digested lignocellulose structures were deduced by comparing the original and digested cell wall HSQC spectra using differential spectra (original – digested) (Fig. 2a) and also by comparing relative signal intensities determined for the major lignin and polysaccharide signals based on their volume integrations (Fig. 2b). Overall, regardless of the lignocellulose diets tested, lignin signals were generally augmented over most of the polysaccharide signals in the digested lignocellulose spectra, which is in line with the chemical analysis data (Table 1) and affirms that polysaccharides were preferentially digested over lignins in the *C. formosanus* digestive system. In the digested J. cedar spectrum, signals from xylan (**X**) and arabinan (**A**) were relatively retained, whereas the signals from glucan (**Gl**), mannans (**M** and **M′**) and glucuronan (**U**) were largely depleted, compared to those in the original J. cedar spectrum. We also found relatively low depletions or even augmentations in the signals from arabinan (**A**) and 2,3 di-*O*-acetyl xylan (**X′″**) in both digested J. beech and rice straw lignocellulose spectra, whereas glucan (**Gl**), non-acetylated (**X**) and mono-acetylated (**X′** and **X″**) xylan, and glucuronan (**U**) signals were clearly depleted. Therefore, apparently, arabinan and highly acetylated xylan residues are relatively well tolerated in the *C. formosanus* digestive system. In the rice straw grass spectra, *p*-coumarate signals (**P**) were remarkably augmented, while in contrast, ferulate signals (**F**) were clearly depleted. Given that *p*-coumarates and ferulates in grass cell walls are mainly attached to lignin and arabinoxylan hemicellulosic backbones, respectively^38^, these data are in a good agreement with our observations of lignin augmentation and hemicellulose depletion in the digested rice straw lignocellulose. Interestingly, however, lignin-bound tricin signals (**T**) were clearly depleted in contrast to the augmented major lignin backbone and lignin-associated *p*-coumarate signals. The result suggests preferential removal of tricin-appended lignin residues in grass lignins, as further demonstrated below.

### 2D NMRs on residual lignins in lignocelluloses digested by *C*. *formosanus* workers

**Figure 3:**
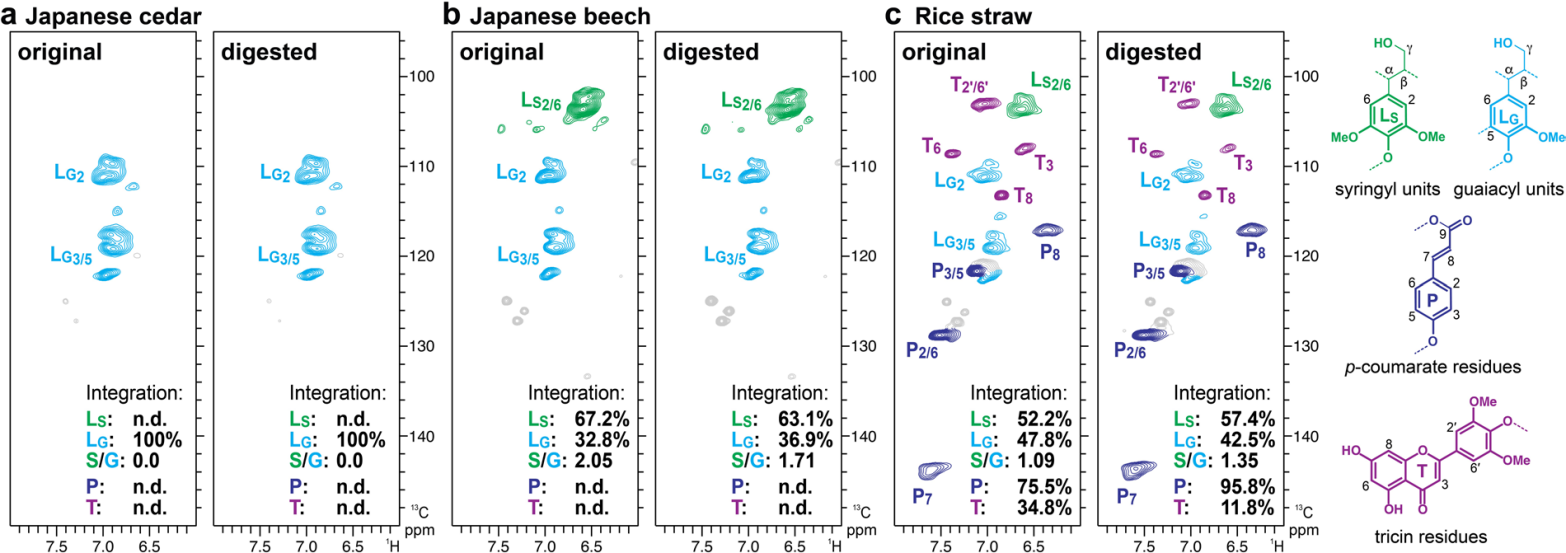
2D ^1^H–^13^C correlation (HSQC) spectra of acetylated samples of lignin-enriched cell walls from original and digested lignocellulose diets fed to *C*. *formosanus* termite workers. Lignin aromatic sub-regions are shown for (**a**) Japanese cedar (softwood), (**b**) Japanese beech (hardwood), and (**c**) rice straw (grass) diets. Volume integrals are given for the major lignin aromatic units that are color-coded to match their assignments in the spectrum. The percentages noted in each spectrum are integrals relative to the sum of the syringyl and guaiacyl lignin unit signals (**L**_**S**_ + **L**_**G**_ = 100%). For signal assignments and structure abbreviations, also see Table S2. n.d., not detected.

**Figure 4:**
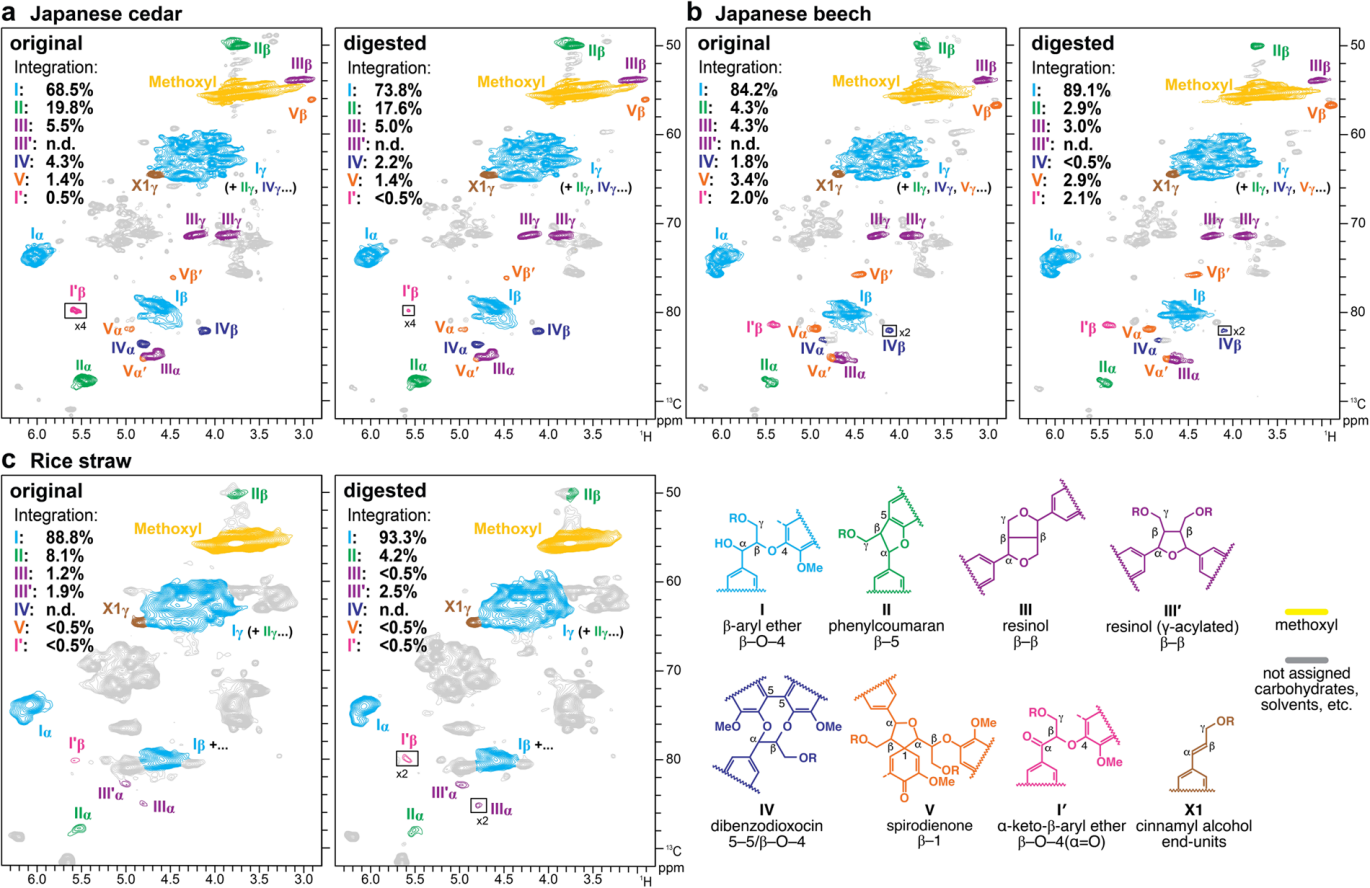
2D ^1^H–^13^C correlation (HSQC) spectra of acetylated samples of lignin-enriched cell walls from original and digested lignocellulose diets fed to *C*. *formosanus* termite workers. Lignin aliphatic-oxygenated sub-regions are shown for (**a**) Japanese cedar (softwood), (**b**) Japanese beech (hardwood), and (**c**) rice straw (grass) diets. Volume integrals are given for the major lignin side-chain structures that are color-coded to match their assignments in the spectrum. The percentages noted in each spectrum are integrals relative to the sum of the annotated lignin side-chain signals (**I** + **I′** + **II + III** + **III′** + **IV** + **V** = 100%). For signal assignments and structure abbreviations, also see Table S2. n.d., not detected.

To more closely investigate possible lignin polymer modifications/decomposition in the termite digestive system, we prepared lignin-enriched cell wall fractions from the original and digested lignocellulose materials via cellulase treatment, which leaves all the lignins and residual polysaccharides^41,42^. The obtained lignin-enriched cell wall fractions were then acetylated to be solubilized in chloroform-*d* for further comprehensive analysis by NMR^35,41,42^. HSQC spectra indicated successful removal of cell wall polysaccharides and concurrent enrichment of lignin polymers and displayed well-resolved signals from major aromatic units as well as various inter-monomeric linkage types in the lignin polymers (Figs 3 and 4, and Table S2).

For the digested J. cedar lignins, the aromatic spectra regions displaying only G unit signals were practically no different from the original lignin spectrum (Fig. 3a). However, the aromatic spectra regions of J. beech and rice straw lignins displayed differences from the corresponding original lignin spectra, and volume integration analysis allowed us to estimate deviations in the aromatic unit distribution patterns between the original and digested lignins (Fig. 3b and c). In the digested J. beech spectrum, syringyl aromatic signals (**L**_**S**_) were slightly depleted along with a concurrent increment in the guaiacyl aromatic signals (**L**_**G**_) compared to those observed in the original lignin spectrum (Fig. 3b). On the other hand, interestingly, the syringyl signals (**L**_**S**_) were conversely augmented with a depletion in guaiacyl signals (**L**_**G**_) in the digested rice straw lignins (Fig. 3c). Syringyl-to-guaiacyl (S/G) ratios estimated by 2D NMR (**L**_**S**_/**L**_**G**_) were 2.05 and 1.71 in the original and digested J. beech lignins, respectively, and 1.09 and 1.35 in the original and digested rice straw lignins, respectively. Such changes of lignin S/G ratios by the termite digestive system were further corroborated by analytical thioacidolysis^43–45^ to determine S/G ratios based on the lignin monomers released by the chemical cleavage of β–*O*–4 lignin substructures; thioacidolysis-derived S/G ratios were 3.05 and 2.37 in the original and digested J. beech lignins, respectively, and 0.84 and 1.31 in the original and digested rice straw lignins, respectively (Table 1). In addition to the change in lignin S/G composition, our NMR data suggested that rice straw lignins were considerably enriched in *p*-coumarate residues, with ~27% proportional increment in the **P**/(**L**_**S**_ + **L**_**G**_) signal ratio, but rather remarkably depleted in tricin residues with ~66% proportional decrease in **T**/(**L**_**S**_ + **L**_**G**_) signal ratio (Fig. 3c). The enrichment in *p*-coumarate residues might be associated with the enrichment of S units in the digested rice straw lignins, since *p*-coumarates in typical grass lignins are attached mainly to S units rather than G units^38^. Taken together, our data provide evidence of partial lignin decomposition in the *C. formosanus* digestive system, where S units in the hardwood J. beech lignins and non-acylated G units and tricin residues in the grass rice straw lignins were preferentially removed.

The aliphatic-oxygenated sub-regions of the HSQC spectra provide information of the major inter-monomeric linkage types in the lignin polymers (Fig. 4, Table S2). Typical lignin linkage signals from β–*O*–4 (**I** and **I′**), β–5 (**II**), β–β (**III** and **III′**), β–*O*–4/5-5 (dibenzodioxocin, **IV**) as well as β–1 (spirodienone, **V**) units were clearly visible in both the original and digested lignin spectra clearly with better resolutions compared to those observed in the whole cell wall spectra (Fig. 2, S1–S3). Volume integration analysis was conducted to clarify differences in the distributions of these inter-monomeric linkage types between the original and digested lignins. Regardless of the three different lignin types tested, the inter-monomeric linkage distribution patterns were found to be overall similar between the original and digested lignins, suggesting that the action of the *C*. *formosanus* digestive system on these major inter-monomeric linkages is not drastic. However, in all the digested lignin spectra, the typical β–*O*–4 unit signals (**I**) were augmented by  5–8% relative to the normalized intensities in the original lignin spectra, mainly at the expense of β–5 (**II**) and resinol-type β–β (**III**) signals. The signals from dibenzodioxin units (**IV**) only seen in the J. cedar and J. beech lignin spectra were also depleted in the digested lignin spectra compared to those observed in the original lignin spectra. Apparently, no clear signal changes were seen for oxidized α-keto-β-aryl ether units (**I′**) and spirodienone (**V**) in J. cedar and J. beech lignins, also for γ-acylated tetrahydrofuran-type β–β (**III′**) in rice straw lignins. Overall, as further discussed below, our data suggest that lignin modification/degradation modes in the termite digestive system vary for different lignin substrates with different chemical structures.

## Discussion

The present data support the view that lignin polymers are at least partially decomposed during their passage through the gut digestive system of lower termites, although, as has been noted by earlier studies^17,19,46^, polysaccharide decomposition clearly exceeds lignin decomposition, and large parts of lignin polymers remain intact in the digestive residues (Table 1, Fig. 2). High-resolution structural data obtained by solution-state 2D HSQC NMR revealed the actions of the *C*. *formosanus* digestive system on the three major lignin types in nature, i.e., softwood, hardwood, and grass lignins.

The aromatic unit composition analysis based on both NMR and thioacidolysis determined that S units are degraded preferentially over G units in the digestion of hardwood J. beech lignins while G units are preferentially degraded over S units in grass rice straw lignins (Fig. 3). These results suggest that lignin modification modes in the termite digestive system may somehow vary for different lignin substrates with different chemical structures. In a finding that supported this, Ke *et al*. reported notably different Py-GC/MS profiles for hardwood (poplar) and grass (barley straw) lignins digested by *C. formosanus*^21^. Both hardwood and grass lignins consist mainly of G and S units, but one of their major structural differences is that the latter is highly γ-acylated by *p*-coumarates, especially on S units^38^. Given that we observed notably augmented *p*-coumarate residues (Fig. 3) as well as relatively unchanged γ-acylated tetrahydrofuran-type β–β linkages (Fig. 4) after the digestion of rice straw lignins, it is conceivable that γ-acylated S units, which are unique and abundant in grass lignins, are relatively tolerable to the lignin processing in the *C*. *fomosanus* digestive system. Another intriguing observation made for the digestion of grass lignins here was that tricin flavonoid units were largely depleted after digestion (Fig. 4). Tricin was recently identified as an authentic lignin monomer that is incorporated into lignin polymers via copolymerization with traditional lignin monomers, i.e., monolignols and their derivatives, upon lignification in grasses including rice^39,40^. Our finding implies that the termite and/or its gut symbionts may possess flavonoid-processing enzymes that preferentially decompose tricin residues in grass lignins. Alternatively, because tricin typically occurs at one terminus of a lignin polymer chain as an end unit^39^, it is also conceivable that tricin-containing oligomeric lignin fragments that were released upon lignin degradation might be preferentially solubilized and removed in the termite digestive system, leaving tricin-less internal lignin polymer units in the insoluble digestive residues.

Previous studies on lignin degradation by lower termites using sensitive Py-GC/MS methods reported pyrograms displaying several phenolic compounds annotated as markers for lignin side-chain oxidation^18–21^. However, our NMR analysis failed to detect any conclusive evidence for such direct oxidation of lignin polymers in the *C*. *formosanus* digestive system: no clear signal increments were observed in the low-field HSQC aromatic regions where signals from oxidized lignin aromatic nuclei typically arise^28,47^, nor were there no clear changes in the relative abundance of oxidized α-keto-β-aryl ether units (**I′**)^28,47^ after termite digestion (Figs 3 and 4). These results suggest that the proposed lignin degradation pathways involving side-chain oxidations could be substantially minor in the *C*. *formosanus* digestive system at least under the tested conditions. Meanwhile, for all the three lignocellulose diets tested, we observed slight increments in the relative abundance of β–*O*–4 and decreased β–5 and resinol-type β–β linkages in the digested lignin polymers (Fig. 4). Li *et al*.^31^ recently reported 2D NMR data obtained for hardwood poplar lignins digested by a fungus-cultivating higher termite, *Odontotermes formosanus*, where they likewise observed relatively enriched β–*O*–4 over β–5 and β–β units in the digested lignins. Collectively, both lower and higher termites and/or their gut symbionts may favor degradations of C–C-bonded lignin inter-monomeric units, e.g., β–5 and β–β, over degradation of ether-bonded β–*O*–4, which somehow contrasts with what has been observed in typical lignin-biodegradation processes undertaken by wood-decaying fungi^27–30^.

Although our present data, along with earlier literature data^18–21,31^, support the view that termites indeed have an ability to partially modify/decompose lignin polymers within their gut digestive systems, there has been no clear evidence that termites can utilize lignin as a nutrient source. In fact, our previous study showed that lignin is non-nutritious or even rather detrimental to *C*. *formosanus* workers when served as a sole food source^33^. Interestingly, however, we recently found that lignin when served with polysaccharides gives marked positive effects on the survival of *C*. *formosanus* workers as well as on their maintenance of hindgut protists, major contributors to the polysaccharide digestion in the digestive system of lower termites, suggesting that the presence of lignin in lignocellulose diet is crucial to maintaining a wholesome hindgut digestive system for efficient polysaccharide conversions^34^. Although further studies are clearly needed to elucidate the mechanisms underlying such dietary effects of lignin, it is plausible that monomeric and/or oligomeric aromatic compounds derived from partial lignin polymer deconstructions may have the beneficial effect of increasing the energy for symbiotic protists, and/or they may trigger positive changes in the bacteria community that also support polysaccharide digestion in the termite digestive system. Profiling gut microbes using advanced genomic techniques may help us clarify the link between the symbiotic community and the dietary effects of lignin. Meanwhile, it is also important to conclusively identify and characterize genes and gene products participating in lignin degradation by the termite host and/or its gut symbionts^9,10,48^. Such studies would provide further insights into the mechanism of lignocellulose biodegradation by wood-feeding insects, which in turn may also contribute to the development of sustainable technologies to utilize lignocellulosic biomass more efficiently.

## Methods

### Termite feeding and sample collection

One thousand two hundred workers of *C. formosanus* Shiraki were starved for 2 days and then allowed to feed in a glass petri dish containing sapwood blocks [20 (R) × 20 (T) × 10 (L) mm] of Japanese cedar (*Cryptomeria japonica*) or Japanese beech (*Fagus crenata*), or 35-mm-long culm straws of rice (*Oryza sativa* L. ssp. *japonica* cv. Nipponbare). Termite feces attached to the surface of each cup were carefully collected every 2 days and pooled over 8 weeks to provide enough materials (ca. 500 mg) for subsequent analysis. The obtained feces samples and pulverized original lignocellulose materials (control) were washed successively with water and 80% ethanol, and then lyophilized to give cell wall residue (CWR) samples used for chemical analysis and NMR spectroscopy. In parallel, fifty workers and five soldiers of *C. formosanus* were subjected to the feeding experiments as described above but on smaller sapwood blocks of Japanese cedar and Japanese beech [10 (R) × 10 (T) × 10 (L) mm] or dried culm straws (25-mm-long)^33,34^. The number of live workers was recorded weekly for 3 weeks. After 3 weeks of feeding, lignocellulose residues were oven-dried and weighed to determine the mass loss during the feeding. Experiments were performed in triplicate. The differences in the survival rate of termites and mass loss of lignocellulose were tested by a IBM SPSS software. Significant differences (*P* < 0.05) between means were calculated by Tukey’s post-hoc test.

### Wet chemistry

Thioglycolic acid lignin assay^33,49^, analytical thioacidolysis^40,44^, and neutral sugar analysis^40,50^, was performed as described previously.

### NMR spectroscopy

For NMR analysis, the CWR samples (ca. 250 mg) prepared as described above were subjected to fine ball-milling as described previously^40^. Aliquots of the ball-milled CWRs (ca. 60 mg) were directly swelled in dimethylsulfoxide (DMSO)-*d*_6_/pyridine-*d*_5_ [4:1 (v/v), 600 μl] for whole-cell-wall NMR analysis^36,37^. The remaining ball-milled CWRs (ca. 190 mg) were further digested with crude cellulases (cellulysin, Calbiochem) and completely acetylated in a DMSO/*N*-methylimidazole/acetic anhydride system as described previously^42^. The obtained acetylated lignin-enriched CWRs were dissolved in 600 μl of chloroform-*d* and subjected to NMR analysis. NMR spectra were acquired on a Bruker Biospin Avance III 800US spectrometer fitted with a cryogenically cooled 5-mm TCI gradient probe. Adiabatic HSQC experiments (“hsqcetgpsp.3”) were performed using the parameters described by Mansfield *et al*.^37^. Data processing used Bruker TopSpin software and the central solvent peaks (DMSO-*d*_6_: δ_C_/δ_H_, 39.5/2.49 ppm; chloroform-*d*: δ_C_/δ_H_, 77.0/7.26 ppm) were used as an internal reference. HSQC plots were obtained with typical matched Gaussian apodization in F2 and squared cosine-bell apodization and one level of linear prediction (16 coefficients) in F1. For constructing differential HSQC spectra and acquiring volume integrations, linear prediction was turned off. For comparison between cell-wall NMR spectra of original and digested lignocelluloses (Fig. 2), major lignin aromatic and polysaccharide anomeric signals (listed in Fig. 2b) were integrated and normalized based on the sum of the integrated signals. For analysis of lignin aromatic composition (Fig. 3), C_2_–H_2_ correlations from **L**_**G**_, C_2_–H_2_/C_6_–H_6_ correlations from **L**_**S**_ and **P**, and C_2′_–H_2′_/C_6′_–H_6′_ correlations from **T** were integrated, and the **L**_**S**_, **P**, and **T** integrals were logically halved. For analysis of lignin inter-monomeric linkage distributions (Fig. 4), well-resolved C_α_–H_α_ contours from **I**, **II**, **III**, **III′**, and **V**, and C_β_–H_β_ contours from **I′** and **VI** were integrated, and **III** and **III′** integrals were logically halved. The relative contour intensities listed in Figs 3 and 4 are expressed on **L**_**S**_ + **L**_**G**_ = 100 and **I** + **I′** + **II** + **III** + **III′** + **IV** + **V** = 100 bases, respectively.

### Data availability

All data necessary to evaluate the conclusions in this study are included in the published paper and its Supplementary Information file. Additional data, if required, will be made available by the corresponding authors upon request.

## Electronic supplementary material

Supplementary Information
