# Supplementary material for: NMR studies on lignocellulose deconstructions in the digestive system of the lower termite Coptotermes formosanus Shiraki
Source: Sci Rep. 2018 Jan 22;8:1290. doi: 10.1038/s41598-018-19562-0 (PMC5778066; doi:10.1038/s41598-018-19562-0)
Supplement: Supplementary file 1 — Supplementary Information [file 41598_2018_19562_MOESM1_ESM.pdf]

Supplementary Information

NMR studies on lignocellulose deconstructions in the digestive system of the lower termite *Coptotermes formosanus* Shiraki

Didi Tarmadi[1,2], Yuki Tobimatsu[1*], Masaomi Yamamura[1], Takuji Miyamoto[1], Yasuyuki Miyagawa[1], Toshiaki Umezawa[1,3] and Tsuyoshi Yoshimura[1*]

[1]Research Institute for Sustainable Humanosphere (RISH), Kyoto University, Gokasho Uji, Kyoto 611-0011, Japan. [2]Research Center for Biomaterials, Indonesian Institute of Sciences (LIPI), Jl. Raya Bogor KM.46, Cibinong, Bogor, West Java, 16911, Indonesia. [3]Research Unit for Development and Global Sustainability, Kyoto University, Gokasho, Uji, Kyoto 611-0011, Japan.

[*]Corresponding authors: Yuki Tobimatsu (ytobimatsu@rish.kyoto-u.ac.jp; Tel: +81-774-38-3626) and Tsuyoshi Yoshimura (tsuyoshi@rish.kyoto-u.ac.jp; Tel: +81-774-38-3664)

Contents:

Table S1. Signal assignments for NMR spectra of whole cell wall samples.

Table S2. Signal assignments for NMR spectra of acetylated lignin-enriched cell wall samples.

Figure S1. 2D HSQC NMR spectra of whole-cell-wall gels from original and digested Japanese cedar (softwood) lignocellulose diets.

Figure S2. 2D HSQC NMR spectra of whole-cell-wall gels from original and digested Japanese beech (hardwood) lignocellulose diets.

Figure S3. 2D HSQC NMR spectra of whole-cell-wall gels from original and digested rice straw (grass) lignocellulose diets.

(Table S1)

Table S1. Assignment of lignin and polysaccharide signals in HSQC spectra of whole cell wall samples.

Labels	δ_C/δ_H (ppm)	Assignment
Lignin signals		
$L_{S2/6}$	104.2/6.77	C_2-H_2 and C_6-H_6 in syringyl units
L_{G2}	111.2/7.06	C_2-H_2 in guaiacyl units
$L_{G5/6}$	114.9/6.78, 115.3/7.02,119.3/6.88	C_5-H_5 and C_6-H_6 in guaiacyl units
I_α	71.5/4.87	C_α-H_α in β-O-4 substructures
$I_{S\beta}$	86.2/4.22	C_β-H_β in β-O-4 substructures linked to syringyl units
$I_{G\beta}$	83.9/4.38	C_β-H_β in β-O-4 substructures linked to guaiacyl units
II_α	87.2/5.50	C_α-H_α in β-5 substructures
II_β	53.6/3.49	C_β-H_β in β-5 substructures
III_α	84.8/4.67	C_α-H_α in resinol-type β-β substructures
III_β	53.7/3.01	C_β-H_β in resinol-type β-β substructures
III_γ	71.1/4.16, 71.1/3.83	C_γ-H_γ in resinol-type β-β substructures
III'_α	82.9/4.98	C_α-H_α in tetrahydrofuran-type β-β substructures
$X1_\gamma$	61.8/4.14	C_γ-H_γ in cinnamyl alcohol end-groups
**Methoxyl**	3.74/55.7	C-H in aromatic methoxyl groups
Polysaccharide signals		
Gl_1	103.5/4.44	C_1-H_1 in (1→4)-β-D-glucopyranosyl units
$Gl_{R1\beta}$	96.9/4.46	C_1-H_1 in (1→4)-β-D-glucopyranosyl units (reducing end)
$Gl_{R1\alpha}$	92.4/5.07	C_1-H_1 in (1→4)-α-D-glucopyranosyl units (reducing end)
X_1	102.1/4.28	C_1-H_1 in (1→4)-β-D-xylopyranosyl units
$X_{R1\beta}$	97.8/4.38	C_1-H_1 in (1→4)-β-D-xylopyranosyl units (reducing end)
$X_{R1\alpha}$	92.5/5.00	C_1-H_1 in (1→4)-α-D-xylopyranosyl units (reducing end)
X'_1	99.9/4.58	C_1-H_1 in 2-*O*-acetyl-β-D-xylopyranosyl units
X'_2	73.6/4.63	C_2-H_2 in 2-*O*-acetyl-β-D-xylopyranosyl units
X''_1	101.8/4.40	C_1-H_1 in 3-*O*-acetyl-β-D-xylopyranosyl units
X''_3	75.1/4.95	C_3-H_3 in 3-*O*-acetyl-β-D-xylopyranosyl units
X'''_1	99.1/4.78	C_1-H_1 in 2,3-di-*O*-acetyl-β-D-xylopyranosyl units
X'''_2	71.2/4.72	C_2-H_2 in 2,3-di-*O*-acetyl-β-D-xylopyranosyl units
X'''_3	74.8/4.94	C_3-H_3 in 2,3-di-*O*-acetyl-β-D-xylopyranosyl units
M_1	100.7/4.64	C_1-H_1 in (1→4)-β-D-mannopyranosyl units
M'_1	99.0/4.86	C_1-H_1 in 2-*O*-acetyl-β-D-mannopyranosyl units
M'_2	70.5/5.30	C_2-H_2 in 2-*O*-acetyl-β-D-mannopyranosyl units
A_1	107.7/5.05, 108.2/4.90	C_1-H_1 in α-L-arabinofuranosyl units
A_2	81.8/3.98	C_2-H_2 in α-L-arabinofuranosyl units
U_1	97.7/5.24	C_1-H_1 in 4-*O*-methyl-α-D-glucuronopyranosyl units
U_4	81.5/3.24	C_4-H_4 in 4-*O*-methyl-α-D-glucuronopyranosyl units
p-Hydroxycinnamate and tricin signals		
$P_{2/6}$	130.1/7.50	C_2-H_2 and C_6-H_6 in *p*-coumarate residues
$P_{3/5}$	115.6/6.85	C_3-H_3 and C_5-H_5 in *p*-coumarate residues
P_7	145.3/7.64	C_7-H_7 in *p*-coumarate residues
P_8	113.8/6.36	C_8-H_8 in *p*-coumarate residues
F_2	111.0/7.36	C_2-H_2 in ferurate residues
F_6	123.2/7.12	C_6-H_6 in ferurate residues
T_3	104.9/7.06	C_3-H_3 in tricin residues
T_6	98.9/6.31	C_6-H_6 in tricin residues
T_8	94.2/6.63	C_8-H_8 in tricin residues
$T_{2'/6'}$	104.3/7.36	$C_{2'}$-$H_{2'}$ and $C_{6'}$-$H_{6'}$ in tricin residues

Measured in DMSO-d_6/Py-d_5 (4:1, v/v). Signal assigment was based on comparison with literature data.[1-5]

(Table S2)

Table S2. Assignment of lignin signals in HSQC spectra of acetylated lignin-enriched cell wall samples.

Labels	δ_C/δ_H (ppm)	Assignment
Aromatic signals		
$L_{S2/6}$	103.7/6.59	C_2-H_2 and C_6-H_6 in syringyl units
L_{G2}	109.8/6.88, 111.2/6.98	C_2-H_2 in guaiacyl units
$L_{G5/6}$	117.6/6.91,119.1/6.88, 122.0/6.95	C_5-H_5 and C_6-H_6 in guaiacyl units
$P_{2/6}$	128.7/7.50	C_2-H_2 and C_6-H_6 in *p*-coumarate residues
$P_{3/5}$	121.7/7.11	C_3-H_3 and C_5-H_5 in *p*-coumarate residues
P_7	143.8/7.59	C_7-H_7 in *p*-coumarate residues
P_8	117.0/6.35	C_8-H_8 in *p*-coumarate residues
T_3	108.0/6.62	C_3-H_3 in tricin residues
T_6	108.8/7.37	C_6-H_6 in tricin residues
T_8	113.2/6.85	C_8-H_8 in tricin residues
$T_{2'/6'}$	103.0/7.05	$C_{2'}$-$H_{2'}$ and $C_{6'}$-$H_{6'}$ in tricin residues
Aliphatic signals		
I_α	73.4/5.97	C_α-H_α in β-O-4 substructures
I_β	79.7/4.60	C_β-H_β in β-O-4 substructures
I'_β	79.8/5.57, 81.4/5.42	C_β-H_β in α-keto-β-O-4 substructures
II_α	87.7/5.45	C_α-H_α in β-5 substructures
II_β	50.1/3.72	C_β-H_β in β-5 substructures
III_α	85.2/4.69	C_α-H_α in resinol-type β-β substructures
III_β	53.8/3.02	C_β-H_β in resinol-type β-β substructures
III_γ	71.6/4.27, 71.6/3.91	C_γ-H_γ in resinol-type β-β substructures
III'_α	82.8/5.00	C_α-H_α in tetrahydrofuran-type β-β substructures
IV_α	83.5/4.82	C_α-H_α in 5-5/β-O-4 (dibenzodioxocin) substructures
IV_β	82.2/4.09	C_β-H_β in 5-5/β-O-4 (dibenzodioxocin) substructures
V_α	81.9/4.95	C_α-H_α in β-1 (spirodienone) substructures
V_β	56.6/2.91	C_β-H_β in β-1 (spirodienone) substructures
$V_{\beta'}$	75.8/4.46	$C_{\beta'}$-$H_{\beta'}$ in β-1 (spirodienone) substructures
$X1_\gamma$	64.4/4.69	C_γ-H_γ in cinnamyl alcohol end-groups
**Methoxyl**	55.5/3.76	C-H in aromatic methoxyl groups

Measured in chloroform-*d*. Signal assigment was based on comparison with literature data.[5-7]

(Supplemental Figure S1)

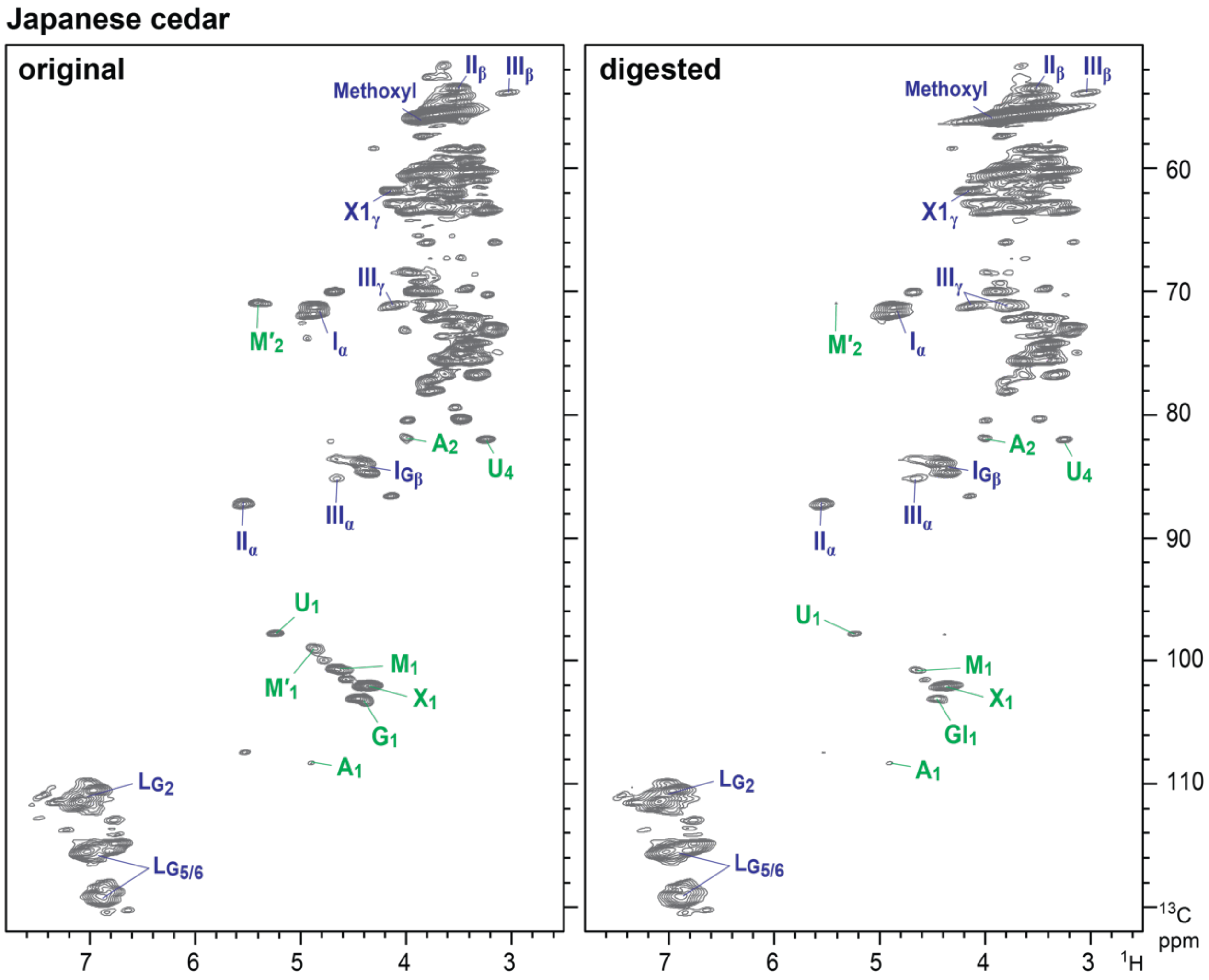

Figure S1. 2D ^{1}H–^{13}C correlation (HSQC) spectra of whole-cell-wall gels from original and digested Japanese cedar (softwood) lignocellulose diets fed to *C. formosanus* termite workers. For signal assignments and structure abbreviations, see Table S1.

(Supplemental Figure S2)

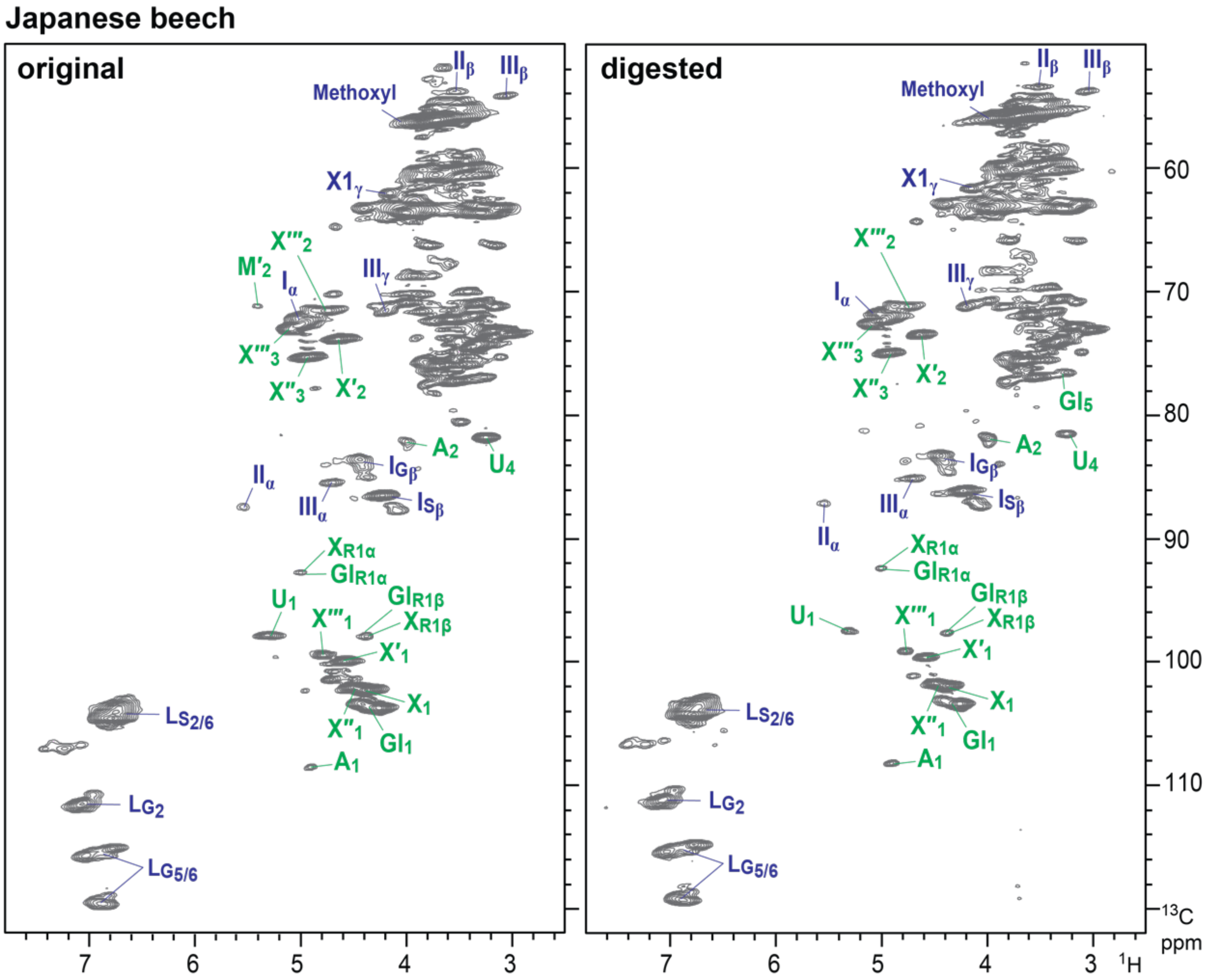

Figure S2. 2D ^{1}H–^{13}C correlation (HSQC) spectra of whole-cell-wall gels from original and digested Japanese beech (hardwood) lignocellulose diets fed to *C. formosanus* termite workers. For signal assignments and structure abbreviations, see Table S1.

(Supplemental Figure S3)

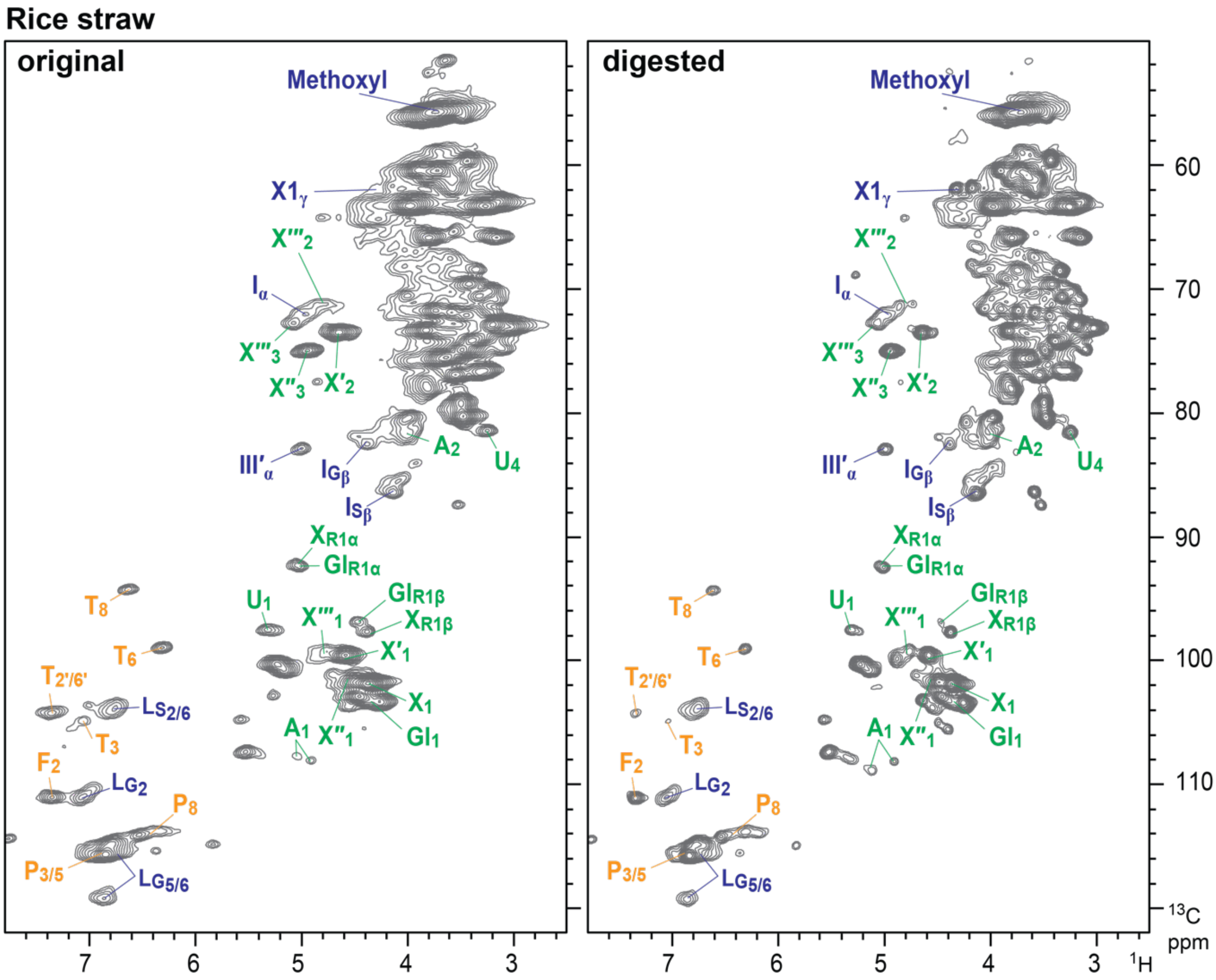

Figure S3. 2D ^{1}H–^{13}C correlation (HSQC) spectra of whole-cell-wall gels from original and digested rice straw (grass) lignocellulose diets fed to *C. formosanus* termite workers. For signal assignments and structure abbreviations, see Table S1.

Supplemental References

1. Kim, H. & Ralph, J. Solution-state 2D NMR of ball-milled plant cell wall gels in DMSO-d_6/pyridine-d_5. *Org. Biomol. Chem.* **8**, 576-591 (2010).

2. Rencoret, J. *et al*. Lignin composition and structure in young versus adult *Eucalyptus globulus* plants. *Plant Physiol.* **155**, 667-682 (2011).

3. Mansfield, S.D., Kim, H., Lu, F. & Ralph, J. Whole plant cell wall characterization using solution-state 2D NMR. *Nat. Protoc.* **7**, 1579-1589 (2012).

4. Brennan, M., McLean, J.P., Altaner, C.M., Ralph, J., & Harris, P.J. Cellulose microfibril angles and cell-wall polymers in different wood types of *Pinus radiata*. *Cellulose*, **19**, 1385-1404 (2012).

5. Lan, W. *et al*. Tricin, a flavonoid monomer in monocot lignification. *Plant Physiol*. **167**, 1284-1295 (2015).

6. Ralph, S.A., Ralph, J., & Landucci, L. NMR database of lignin and cell wall model compounds. URL:https://www.glbrc.org/databases_and_software/nmrdatabase/NMR_DataBase_2009_Complete.pdf.

7. Stewart, J.J., Akiyama, T., Chapple, C., Ralph, J. & Mansfield, S.D. The effects on lignin structure of overexpression of ferulate 5-hydroxylase in hybrid poplar. *Plant Physiol.* **150**, 621–635 (2009).
